# Plant-Based Biomaterials as Bio-Instructive Immunomodulators: Design Principles, Mechanisms, and Translational Challenges

**DOI:** 10.3390/life16040538

**Published:** 2026-03-24

**Authors:** Stefania Lamponi

**Affiliations:** 1 Department of Biotechnology, Chemistry & Pharmacy, University of Siena, Via Aldo Moro 2, 53100 Siena, Italy; stefania.lamponi@unisi.it; 2 SienabioACTIVE, University of Siena, Via Aldo Moro 2, 53100 Siena, Italy

**Keywords:** plant-based biomaterials, immunomodulation, macrophage polarization, polysaccharides, polyphenols, hydrogels, tissue engineering, clinical translation, sustainability, extracellular vesicles

## Abstract

Plant-based biomaterials are increasingly recognized as bio-instructive platforms capable of actively modulating immune responses rather than functioning solely as passive structural supports. In this context, the term plant-based refers to photosynthetic biomass-derived platforms, including both terrestrial plants and marine macroalgae, reflecting their shared richness in polysaccharides and secondary metabolites relevant to immune engineering and regenerative medicine. This review critically synthesizes current evidence on plant-derived polysaccharides and phytochemicals, including algal sulfated polysaccharides (fucoidan, alginate, carrageenan, and ulvan), terrestrial plant polysaccharides (e.g., *Lycium barbarum* and *Aloe vera* derivatives), polyphenols, and other secondary metabolites such as terpenoids and alkaloids, highlighting their roles as immunomodulators in biomedical contexts. Key mechanisms include macrophage polarization along an M1–M2 continuum, pattern recognition receptor engagement, redox and metabolic regulation, and crosstalk between innate and adaptive immunity, with emphasis on context-dependent signaling and structural heterogeneity. Material design parameters, including molecular weight and chemical functionalization, are critical determinants of immune responses. Advanced delivery systems, such as hydrogels, nanocomposites, phytosomes, and plant-derived extracellular vesicles (EVs), enable improved stability and spatiotemporal control. Applications in wound and musculoskeletal regeneration are discussed alongside translational challenges, including variability, reproducibility, regulatory issues, and the need for standardized characterization and immune validation.

## 1. Introduction

### 1.1. Historical Context and Evolution of Biomaterials Design

Since the emergence of biomaterials science in the 1960s, the field has undergone substantial conceptual and technological evolution. Early, first-generation biomaterials were primarily designed to fulfill mechanical and structural requirements, such as strength, stability, and basic biocompatibility, while the host immune response was largely regarded as an undesirable side effect to be minimized or avoided [1]. Subsequent second-generation biomaterials showed improved surface chemistry, porosity, and degradation profiles to reduce inflammatory reactions and enhance tissue integration.

Advances in immunology and regenerative biology have since driven a paradigm shift toward third-generation bioactive or “smart” biomaterials that actively interact with biological systems and guide cellular behavior. Rather than aiming for immune inertness, contemporary biomaterial design increasingly recognizes immune cells as central regulators of tissue repair and regeneration. In this context, inflammation is no longer viewed solely as a pathological process, but as a dynamic and essential biological signal that must be precisely regulated in space and time to promote functional healing rather than chronic damage [2].

Within this framework, biomaterials capable of bio-instruction, which are materials that actively direct immune cell phenotype and function, have gained considerable attention. Plant-based biomaterials have emerged as particularly promising candidates for such immune-engineering strategies, owing to their intrinsic bioactivity, chemical diversity, and long evolutionary history of interaction with biological defense systems [3,4].

### 1.2. Why Plants? Evolutionary and Functional Rationale for Plant-Based Bioactive Molecules

Plants are continuously exposed to environmental stressors, including pathogenic microorganisms, parasites, and herbivores, and have consequently evolved a broad repertoire of structurally diverse secondary metabolites and polysaccharides with biological activity [5,6]. These compounds function as components of complex defense and signaling systems, many of which interact with conserved immune pathways shared across biological kingdoms [7,8]. As a result, plant-derived molecules often display immunomodulatory properties that are directly relevant to mammalian immune regulation [9].

In contrast to many synthetic compounds designed through target-specific rational drug discovery, plant-derived bioactive molecules frequently engage multiple molecular targets simultaneously [10]. This multi-target mode of action can confer greater biological robustness and may reduce the likelihood of resistance development, particularly in inflammatory and infectious contexts [11,12]. Such pleiotropic activity is increasingly recognized as advantageous in complex biological processes such as tissue regeneration, where coordinated modulation of multiple immune and cellular pathways is required [13].

Ethnopharmacology further provides an important conceptual and practical foundation for plant-based biomedical research [14]. The traditional use of medicinal plants, refined through centuries of empirical observation, offers valuable prioritization cues for bioactive compounds with therapeutic potential [15]. When integrated with modern analytical, molecular, and materials science approaches, ethnopharmacological knowledge represents a complementary strategy for high-throughput screening, enabling more informed selection of candidates for biomaterial development and translational investigation [16]. Accordingly, throughout this review we adopt an operational definition of plant-based materials that includes macroalgae as established biomedical sources of structurally unique polysaccharides [17,18].

### 1.3. Plant-Derived Biomaterials in the Context of Conventional Biomaterial Classes

From a biomaterials perspective, plant-derived systems also occupy a distinctive position relative to other widely used material classes in regenerative medicine. Synthetic polymers such as poly(lactic-co-glycolic acid) (PLGA), polyethylene glycol (PEG), polyvinyl alcohol (PVA), and polyvinyl pyrrolidone (PVP) are extensively used because their physicochemical properties, degradation profiles, and mechanical behavior can be precisely tuned during synthesis. However, these materials are generally considered biologically inert, and immunomodulatory activity often requires additional functionalization or incorporation of bioactive molecules [19,20,21].

Animal-derived matrices, including collagen, gelatin, and decellularized extracellular matrix (ECM), provide excellent biological compatibility and structural similarity to native tissues and have therefore been widely explored as scaffolds for regenerative applications. Nevertheless, their use may present limitations related to batch-to-batch variability, potential immunogenicity, risk of pathogen transmission, and ethical concerns associated with animal sourcing [22,23].

In comparison, plant-derived biomaterials offer a complementary approach. Many plant polysaccharides and phytochemicals exhibit intrinsic immunomodulatory activity, enabling direct interaction with pattern recognition receptors and redox-sensitive signaling pathways without extensive chemical modification. In addition, plant-based materials generally present lower zoonotic risk and can often be produced through more sustainable and scalable supply chains. At the same time, challenges related to structural heterogeneity and standardization remain important considerations for their translation into clinical biomaterials [17,18].

### 1.4. Scope and Organization of This Review

Unlike previous reviews that predominantly address structural properties or isolated pharmacological effects, the present work integrates materials design, immune signaling mechanisms, and translational constraints within a unified immune-engineering framework. It examines plant-based biomaterials as bio-instructive immunomodulators, with particular emphasis on their structural determinants, molecular mechanisms of immune regulation, and pathways toward clinical translation. For conceptual clarity, this review adopts a functional definition of plant-based biomaterials that includes both terrestrial plants and marine macroalgae as photosynthetic biomass-derived systems.

The review is organized around six interconnected themes: (1) structural characteristics and bioactive properties of plant-derived polysaccharides and phytochemicals; (2) molecular and cellular mechanisms of immunomodulation, including regulation of innate and adaptive immune responses; (3) tissue-specific applications in regenerative medicine and tissue engineering; (4) advanced fabrication and delivery strategies designed to enhance stability, bioavailability, and functional performance; (5) translational pathways, including standardization, regulatory, and ethical considerations; (6) critical research gaps and future directions.

The primary focus is placed on plant-derived polysaccharides, phytochemicals, and plant-derived extracellular vesicle-like nanoparticles as immunomodulatory biomaterials. Plant-tissue-derived decellularized scaffolds (e.g., leaf vasculature templates) and nanocellulose-based structural platforms are not addressed in detail, as their principal function is architectural rather than immunoregulatory. Although these systems represent significant advances in bio-fabrication, the present review specifically emphasizes plant-derived materials that actively modulate innate and adaptive immune responses. This defined scope enables a mechanistically grounded and translationally oriented analysis of immune-instructive plant-based biomaterials, while integrating perspectives from materials science, immunology, sustainability, and ethical resource stewardship.

## 2. Natural Plant-Derived Polysaccharides: Structural Diversity, Bioactive Properties, and Therapeutic Applications

Plant-derived polysaccharides constitute one of the most extensively explored classes of biomaterials in regenerative medicine and immuneengineering [24,25]. Their structural diversity, intrinsic biocompatibility, and capacity to engage conserved immune recognition pathways render them particularly suitable as bio-instructive materials [26]. Unlike inert synthetic polymers, many plant polysaccharides possess inherent biological activity, enabling direct modulation of immune cell behavior while simultaneously providing mechanical support and delivery functionality [27,28].

From an immunological perspective, polysaccharides derived from terrestrial plants and marine macroalgae share common features, high molecular weight, repeating carbohydrate motifs, and frequent chemical substitutions (e.g., sulfation, acetylation), that facilitate interaction with pattern recognition receptors and downstream immune signaling cascades [29,30,31]. This section examines major classes of marine- and terrestrial-derived polysaccharides, emphasizing how structural characteristics govern immunomodulatory function and translational potential [32].

### 2.1. Marine Polysaccharides: Seaweed-Derived Compounds

Marine macroalgae represents a rich and relatively underexploited source of structurally unique polysaccharides with high biomedical relevance [33,34]. The aqueous environments and evolutionary pressures experienced by seaweed have favored the development of sulfated and highly charged polysaccharides with pronounced bioactivity, many of which exhibit immunomodulatory, antioxidant, and antimicrobial properties [35,36].

#### 2.1.1. Fucoidan: Multifunctional Sulfated Polysaccharide with Translational Potential

Fucoidan is among the most intensively investigated sulfated polysaccharides derived from brown macroalgae (*Phaeophyceae*) [37]. It has attracted considerable interest due to its favorable biocompatibility profile and wide-ranging biological activities, including immunomodulation, anti-inflammatory effects, antioxidant activity, and support of tissue repair processes [35,38,39,40]. Fucoidan has achieved regulatory acceptance in specific non-pharmaceutical contexts (e.g., food and nutraceutical applications), while its development as a clinical-grade biomaterial or therapeutic agent remains an active area of translational research [41].

Structurally, fucoidan consists primarily of α-L-fucose residues linked through glycosidic bonds, with varying degrees of sulfation, acetylation, and branching depending on algal species, geographic origin, and extraction methodology [29,40]. This intrinsic structural heterogeneity underlies both its biological versatility and its translational challenges. Variations in sulfation pattern, molecular weight distribution, and monosaccharide composition critically influence receptor engagement and downstream immune signaling. In particular, the position of sulfate groups (e.g., C-2 versus C-4 positions of fucose residues) modulates affinity for selectins and TLR-mediated pathways, thereby altering leukocyte recruitment and macrophage activation profiles. These observations further support a structure–activity relationship [42,43].

Molecular weight plays a particularly significant role in determining bioactivity. Low-molecular-weight fucoidan fractions generally demonstrate enhanced cellular uptake and more pronounced anti-inflammatory effects, whereas higher-molecular-weight preparations exhibit superior rheological properties and are better suited for hydrogel formation and sustained-release applications [44,45]. These properties make fucoidan especially attractive for incorporation into polysaccharide-based hydrogels designed for immunomodulatory wound dressings or regenerative scaffolds [46].

Mechanistically, fucoidan modulates immune responses through multiple pathways, including suppression of pro-inflammatory cytokine production, regulation of macrophage polarization, and attenuation of oxidative stress [47,48]. However, clinical translation remains constrained by several factors: batch-to-batch variability linked to biological source material, incomplete structure–activity relationship mapping, and challenges in achieving reproducible chemical modification and crosslinking under good manufacturing practice conditions [49]. Despite these limitations, fucoidan remains a benchmark candidate for plant-based immunomodulatory biomaterials [50].

#### 2.1.2. Alginate, Carrageenan and Ulvan: Structurally Robust Polysaccharides with Distinct Immunological Roles

Alginate, extracted from brown macroalgae via mild alkaline processes, is widely used in biomedical engineering due to its biocompatibility, biodegradability, and ease of processing into hydrogels, microspheres, fibers, and porous scaffolds [51,52]. Alginate gels form rapidly under physiological conditions through ionic crosslinking with divalent cations, most commonly calcium, enabling encapsulation of cells and labile biomolecules [53,54].

While alginate is often regarded as a structurally inert material, increasing evidence suggests that its molecular architecture and purity significantly influence host immune responses [19,54]. Residual contaminants, molecular weight distribution, and block composition (mannuronic vs. guluronic acid content) affect macrophage activation and fibrotic responses, underscoring the importance of material purification and characterization [55]. These findings emphasize that alginate bioactivity is not intrinsic but emerges from defined structural and compositional parameters. In particular, the mannuronic-to-guluronic (M/G) ratio, molecular weight distribution, and endotoxin contamination critically determine Toll-Like Receptor (TLR)-mediated responses and fibrotic outcomes. This context-dependent behavior underscores a structure–activity relationship in which purification level and molecular architecture dictate immunological performance. Thus, alginate serves as an instructive example of how ostensibly “passive” polysaccharides can exert immunological effects depending on structural and processing parameters.

Carrageenan, derived from red macroalgae (Rhodophyceae), consists of sulfated galactans with repeating units of galactose and 3,6-anhydrogalactose [56]. Unlike alginate, carrageenan exhibits intrinsic immunomodulatory activity, largely attributable to its sulfation pattern [57]. Different carrageenan types (κ-, ι-, and λ-carrageenan) display distinct biological effects, influencing inflammatory signaling, antiviral activity, and immune cell recruitment [58,59]. The immunological activity of carrageenan highlights the central role of sulfate ester positioning and density in determining receptor engagement and downstream immune responses.

Ulvan, a sulfated heteropolysaccharide isolated from green macroalgae (*Chlorophyta*), further expands the functional diversity of marine polysaccharides [60]. Composed of rhamnose, xylose, glucuronic acid, and iduronic acid residues, ulvan demonstrates immunomodulatory activity through interactions with pattern recognition receptors and adhesion molecules [61,62]. Its dual capacity to provide structural support and bioactive signaling positions ulvan as a promising candidate for next-generation immunoactive scaffolds [63].

### 2.2. Terrestrial Plant Polysaccharides: Traditional Knowledge and Modern Immune Engineering

Terrestrial plants have served as sources of medicinal polysaccharides for centuries, long before the molecular basis of immune regulation was understood [64]. Contemporary research has begun to elucidate how these polysaccharides influence innate and adaptive immunity, providing mechanistic insight into their long-standing therapeutic use [65].

#### 2.2.1. *Lycium barbarum* Polysaccharides: Immunomodulation and Microbiota Interactions

*Lycium barbarum* (goji berry) is among the most extensively studied medicinal plants in traditional Asian medicine, with documented use spanning more than two millennia [66]. Its bioactive polysaccharides (*Lycium barbarum* polysaccharides LBP) comprise heterogeneous mixtures of neutral, acidic, and protein-bound polysaccharides with pronounced immunomodulatory activity [67].

LBP enhances immune function through multiple mechanisms, including activation of macrophages, stimulation of lymphocyte proliferation, enhancement of natural killer cell cytotoxicity, and promotion of antibody production [68,69,70]. Beyond direct immune activation, LBP influences adaptive immunity by modulating regulatory T cell differentiation and suppressing excessive pro-inflammatory signaling in disease models.

A particularly important and emerging aspect of LBP activity is its role as a prebiotic immunomodulator. As indigestible polysaccharides, LBP selectively promotes the growth of beneficial gut microbiota, including Lactobacillus and Bifidobacterium species [71]. Microbiota-derived metabolites subsequently influence systemic immune regulation, linking intestinal polysaccharide exposure to distal immune effects [72]. This indirect immunomodulatory pathway is increasingly recognized as relevant for biomaterials intended for long-term implantation or oral delivery.

Despite promising biological effects, clinical translation of LBP is hindered by substantial variability in extraction protocols, plant cultivars, and processing conditions, resulting in inconsistent polysaccharide composition and bioactivity [73]. Standardization remains a critical unmet requirement.

#### 2.2.2. Acemannan from *Aloe vera*: Structure-Dependent Immunoregulation

Aloe vera has been used medicinally across diverse cultures, with acemannan identified as its principal immunologically active polysaccharide [74]. Acemannan is a β-(1→4)-linked polymannose with variable acetylation, a feature that critically determines its biological function [75].

Acemannan exhibits broad bioactivity, including stimulation of macrophage activation, promotion of wound healing through fibroblast proliferation and collagen synthesis, antioxidant activity, and modulation of inflammatory signaling pathways [76,77,78]. Experimental studies indicate that partially acetylated acemannan exhibits greater immunomodulatory potency than fully acetylated or deacetylated forms, underscoring the importance of structure–activity relationships [79]. Specifically, the degree of acetylation directly influences receptor interaction and macrophage activation, with partially acetylated forms exhibiting enhanced immunostimulatory activity. This provides a clear example of structure–activity relationship-driven immunomodulation, where controlled chemical modification can be leveraged to fine-tune biological responses. Mechanistically, acemannan interacts with pattern recognition receptors, including TLR, triggering downstream signaling pathways involved in immune activation and tissue repair [80]. These properties make acemannan a compelling example of how subtle chemical features can translate into pronounced immunological effects.

### 2.3. Structural Determinants and Chemical Modification of Plant Polysaccharides

#### 2.3.1. Physicochemical Parameters Governing Bioactivity

The biological performance of plant-derived polysaccharides is dictated by a combination of chemical composition, molecular architecture, and higher-order structure. Key determinants include molecular weight, degree and pattern of substitution (e.g., sulfation, acetylation), charge density, solubility, and three-dimensional conformation. Small variations in these parameters can dramatically alter immune cell engagement and polarization outcomes [81]. These effects are not merely quantitative but highly structure-dependent. In particular, molecular weight distribution, degree and regioselectivity of substitution, and charge density collectively determine pattern recognition receptor engagement and downstream signaling pathways. For example, sulfation pattern and position along the polysaccharide backbone critically influence binding affinity to TLR2/4 and selectins, thereby modulating macrophage polarization and cytokine profiles. This highlights a clear structure–activity relationship, whereby even subtle structural variations can shift immune responses between pro-inflammatory and pro-reparative phenotypes. These considerations further reinforce the importance of structure–activity relationships in immunomodulatory contexts [66]. Importantly, not only the overall degree of sulfation but also the specific position of sulfate groups along the polysaccharide backbone can strongly influence receptor recognition and immune signaling. Studies on marine sulfated polysaccharides have shown that positional sulfation of fucose residues in fucoidan, particularly at the C-2 or C-4 positions, can alter the affinity of the polymer for adhesion molecules such as P-selectin and L-selectin, which regulate leukocyte recruitment during inflammatory responses [82,83]. Similarly, variations in sulfation patterns have been reported to influence the interaction of sulfated polysaccharides with TLR2 and TLR4, thereby affecting macrophage activation and cytokine secretion profiles [84]. These observations highlight that even subtle structural differences, including regioselective sulfation, may lead to markedly different immunological outcomes. Consequently, detailed structural characterization of plant- and algae-derived polysaccharides, including sulfation pattern, molecular weight distribution, and branching architecture, remains essential for establishing reproducible structure–activity relationships and for guiding rational biomaterial design. This sensitivity is also evident in plant pectic polysaccharides, where immune stimulation can depend strongly on high molecular weight and specific structural domains [85].

Despite increasing recognition of structure–function relationships, quantitative and reproducible mapping between defined structural parameters and specific immune outcomes remains underdeveloped across most plant-derived systems. Bridging this gap will require standardized molecular profiling combined with time-resolved and multiparametric immune assays.

Advanced analytical techniques are essential to resolve structure–activity relationships in plant-derived polysaccharides. Nuclear magnetic resonance (NMR) spectroscopy enables detailed characterization of substitution patterns and linkage structures, while size-exclusion chromatography coupled with multi-angle light scattering (SEC-MALS) provides accurate molecular weight distribution profiles. Mass spectrometry (MS)-based approaches further support compositional and structural analysis. Integration of these techniques is critical to correlate defined molecular features with immune outcomes and to ensure reproducibility across studies [86].

#### 2.3.2. Chemical Modification Strategies for Immune Engineering

Chemical modification enables precise tuning of polysaccharide bioactivity and material performance. Oxidation reactions introduce aldehyde groups that facilitate dynamic covalent crosslinking via Schiff base formation, supporting injectable and self-healing hydrogel systems [87,88,89]. Carboxymethylation enhances hydrophilicity and provides conjugation sites for bioactive molecules [90]. Sulfation can increase immunological activity through enhanced pattern recognition receptor engagement, while acetylation can modulate physicochemical behavior and functional performance in delivery/biomedical settings [66,91]. Bioconjugation strategies, including click chemistry and other bio-orthogonal approaches, allow covalent attachment of growth factors, antimicrobial peptides, or small-molecule drugs while preserving biological function [92,93,94,95]. Together, these approaches enable rational design of plant-based polysaccharide biomaterials with predictable and tunable immunological outcomes. A comparative overview of representative plant-based biomaterials, highlighting structural determinants, Pattern Recognition Receptor (PRR) involvement, and immunological effects, is provided in Table 1 which includes also selected phytochemicals discussed in Section 3.

Comparative overview of representative plant-derived polysaccharides, phytochemicals, and extracellular vesicle-like nanoparticles, highlighting key structural determinants, pattern recognition receptor (PRR) involvement, dominant immune effects, level of preclinical or clinical evidence, and selected supporting references. The table emphasizes structure–function relationships and context-dependent immunomodulatory outcomes relevant to regenerative medicine and immune engineering.

### 2.4. Structural Plant Polysaccharides with Emerging Immunomodulatory Roles

In addition to the polysaccharides discussed above, several plant-derived structural polymers traditionally used for their mechanical or rheological properties are increasingly recognized for their potential immunomodulatory effects. Among these, cellulose, starch, and pectin represent some of the most abundant plant biopolymers and are widely explored in biomedical materials and tissue engineering.

Cellulose, the primary structural component of plant cell walls, has been extensively investigated as a scaffold material due to its mechanical strength, biocompatibility, and versatility in chemical modification. In its nanostructured forms, including nanocellulose and bacterial cellulose composites, cellulose-based materials have demonstrated the ability to influence macrophage adhesion, cytokine secretion, and inflammatory signaling pathways [106,107]. These properties have stimulated growing interest in cellulose-based scaffolds for wound healing and soft tissue regeneration.

Starch, another abundant plant polysaccharide composed primarily of amylose and amylopectin, has also attracted attention as a biodegradable biomaterial. Modified starch derivatives and starch-based hydrogels have shown the capacity to modulate inflammatory responses and support tissue repair, particularly when combined with other bioactive polymers or nanoparticles [108]. In regenerative medicine contexts, starch-based materials can provide both structural support and controlled release of bioactive molecules.

Pectin, a complex heteropolysaccharide rich in galacturonic acid residues, has also demonstrated emerging immunomodulatory potential. Pectic polysaccharides can interact with immune receptors and influence inflammatory signaling pathways, including TLR-mediated responses and macrophage activation [109,110]. Recent studies have suggested that pectin-derived materials may contribute to the regulation of intestinal immune homeostasis and inflammatory responses, highlighting their broader potential as immunomodulatory biomaterials.

Taken together, these structural plant polysaccharides illustrate that materials historically regarded as passive structural components may also exert biologically relevant immune effects depending on their molecular structure, purity, and processing conditions.

## 3. Plant-Based Phytochemicals and Bioactive Secondary Metabolites: Molecular Mechanisms in Immune Regulation

Beyond structural polysaccharides, plants produce a wide array of low-molecular-weight secondary metabolites that play central roles in immune modulation and tissue regeneration [111,112]. These phytochemicals, particularly polyphenols, flavonoids, terpenoids, alkaloids, and lectins, have evolved as components of plant defense systems and signaling networks [5]. When integrated into biomaterial platforms, they act as potent bioactive cues capable of directing immune cell behavior, regulating oxidative stress, and shaping regenerative microenvironments [113].

In contrast to polysaccharides, which often exert effects through pattern recognition receptor engagement and structural bio-instruction, phytochemicals primarily function through modulation of intracellular signaling pathways, transcriptional programs, and redox balance [114]. Their combination with plant-based scaffolds therefore enables multiscale immunomodulation, spanning extracellular recognition to intracellular signal integration [115].

### 3.1. Polyphenolic Compounds: From Plant Defense to Therapeutic Agents

Polyphenols constitute one of the most extensively studied classes of plant secondary metabolites due to their pronounced antioxidant, anti-inflammatory, and immunomodulatory properties [116,117]. Structurally, polyphenols are characterized by multiple phenolic hydroxyl groups, which confer both chemical reactivity and biological versatility. In mammalian systems, these compounds influence immune regulation by targeting signaling pathways, transcription factors, and metabolic processes central to inflammation and tissue repair [96].

#### 3.1.1. Polyphenols in Bone Biology and Immunoregulation

Bone regeneration is increasingly recognized as an immune-regulated process, in which osteogenesis, angiogenesis, and inflammation are tightly coupled [118]. Polyphenolic compounds, including flavonoids, phenolic acids, and condensed tannins, modulate bone homeostasis through coordinated actions on osteoblasts, osteoclasts, endothelial cells, and immune populations collectively governing osteoimmunology [97].

Polyphenols promote osteoblast differentiation and function primarily through activation of the Wnt/β-catenin signaling pathway and upregulation of osteogenic transcription factors such as Runx2 and Osterix [119]. Concurrently, they suppress osteoclast genesis by inhibiting receptor activator of nuclear factor κB ligand (RANKL) signaling and reducing the production of pro-osteoclastic cytokines, including Tumor Necrosis Factor (TNF)-α and IL-6 [120].

Beyond direct effects on bone cells, polyphenols regulate immune-mediated bone remodeling by attenuating chronic inflammation and oxidative stress [121]. By scavenging reactive oxygen species and modulating immune cytokine profiles, polyphenols restore a regenerative milieu conducive to bone repair [122].

Recent advances in biomaterial-assisted delivery have addressed key limitations of polyphenols, particularly their poor aqueous solubility and rapid metabolism [94]. Encapsulation within hydrogels, nanoparticles, and surface-functionalized scaffolds improves stability, local retention, and tissue-specific delivery [123]. Despite strong preclinical evidence, however, translation into clinical bone therapies remains limited [124].

#### 3.1.2. Curcumin: A Model Polyphenol for Immunomodulatory Biomaterials

Curcumin, the principal bioactive component of Curcuma longa, exemplifies both the therapeutic potential and translational challenges of polyphenolic phytochemicals [98]. Curcumin exerts potent anti-inflammatory effects through inhibition of NF-κB signaling, suppression of pro-inflammatory cytokine production (TNF-α, Interleuchin (IL)-1β, IL-6), downregulation of Cyclooxygenase-2 (COX-2) and inducible nitric oxide synthase, and attenuation of immune cell infiltration at inflammatory sites [125,126].

In preclinical cancer and inflammatory disease models, curcumin demonstrates multifaceted bioactivity, including apoptosis induction, inhibition of angiogenesis, suppression of epithelial–mesenchymal transition, and modulation of both innate and adaptive immune responses [127]. When incorporated into plant-based biomaterial systems, curcumin provides synergistic antimicrobial, antioxidative, and immunomodulatory effects [99].

A major barrier to curcumin translation is its extremely low oral bioavailability [128]. Advanced formulation strategies, including lipid-based carriers, polymeric nanoparticles, hydrogel matrices, and phytosome technology, substantially enhance bioavailability and prolong therapeutic action [100,129,130]. A comparative overview including curcumin is provided in Table 1.

#### 3.1.3. Icariin: A Multifunctional Flavonoid for Regenerative Immunomodulation

Icariin, a prenylated flavonol isolated from Epimedium species, has emerged as a promising bioactive molecule for regenerative medicine [131]. Icariin promotes osteogenic differentiation of mesenchymal stem cells through activation of AKT and Wnt/β-catenin signaling, while concurrently suppressing osteoclast activity by downregulating RANKL and macrophage colony-stimulating factor expression [132,133].

Importantly, icariin exerts pleiotropic protective effects, including cardioprotective, hepatoprotective, nephroprotective, and neuroprotective activities. However, its clinical translation is limited by poor bioavailability and rapid clearance, motivating the development of nano-formulations and biomaterial-based delivery systems [134].

### 3.2. Plant Lectins: Carbohydrate-Binding Proteins as Immune Recognition Modules

Plant lectins are carbohydrate-binding proteins that recognize specific glycan motifs without enzymatic modification [135]. In biomedical contexts, lectins modulate immune responses through engagement of lectin receptors, complement activation, and regulation of antigen presentation [136]. When incorporated into biomaterial platforms, lectins function as immune-targeting ligands enabling selective interaction with innate immune cells [137].

### 3.3. Phytochemicals in Complex Wound Repair: Integrated Immunomodulatory Pathways

#### 3.3.1. Multicomponent Regulation of Chronic and Diabetic Wounds

Chronic and diabetic wounds are characterized by persistent inflammation, impaired angiogenesis, metabolic dysregulation, and infection susceptibility [138]. Plant-derived phytochemicals address these challenges through simultaneous modulation of inflammatory signaling, macrophage polarization, angiogenesis, and oxidative stress [139].

#### 3.3.2. Reactive Oxygen Species Modulation as a Central Therapeutic Axis

**Figure 1:**
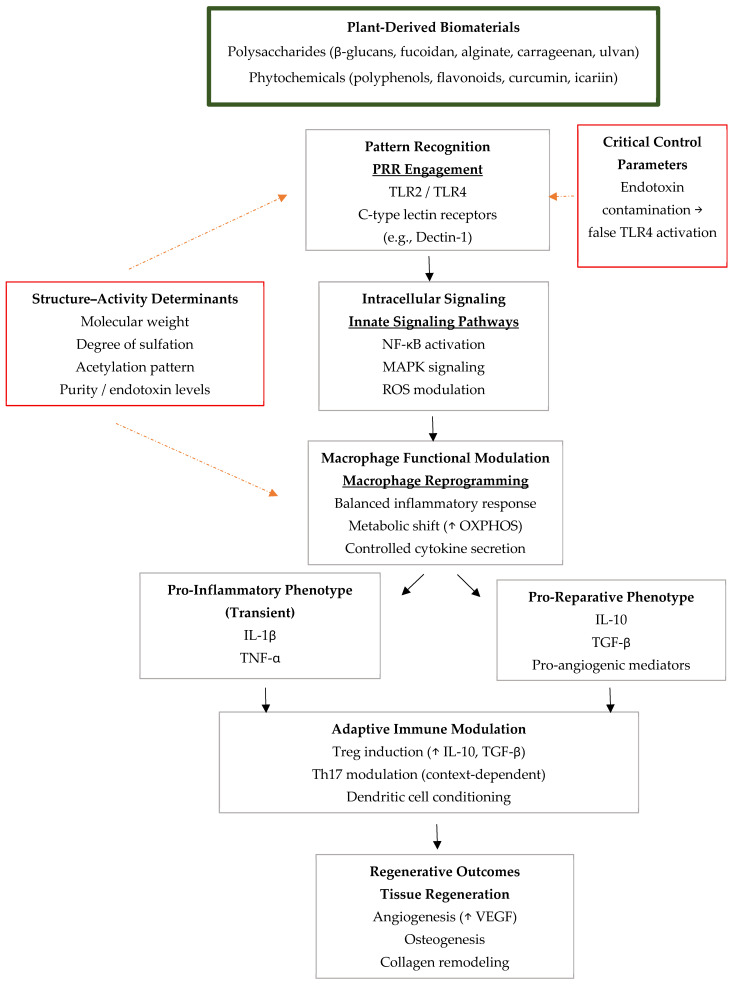
Immune-instructive mechanisms of plant-derived biomaterials in regenerative contexts.

Reactive oxygen species play a dual role in wound healing, acting as signaling mediators at physiological levels but driving chronic inflammation when excessive [140]. Plant-derived compounds mitigate pathological Reactive Oxygen Species (ROS) accumulation through direct scavenging, activation of endogenous antioxidant pathways (notably Nrf2 signaling), and suppression of ROS-driven inflammatory cascades [141,142]. The coordinated innate and adaptive immune mechanisms through which plant-derived biomaterials shape regenerative microenvironments are summarized in Figure 1.

Schematic overview of the molecular and cellular pathways through which plant-derived biomaterials modulate innate and adaptive immune responses. Plant polysaccharides, phytochemicals, and plant-derived extracellular vesicle-like nanoparticles engage pattern recognition receptors (PRRs), including TLR2, TLR4, and C-type lectin receptors (e.g., Dectin-1), triggering intracellular signaling pathways such as Nuclear Factor kappa-light-chain-enhancer of activated B cells (NF-κB) activation, Mitogen-Activated Protein Kinase (MAPK) signaling, redox modulation, and Nuclear factor erythroid 2-related factor 2 (NRF2) activation. These signaling events promote macrophage reprogramming, characterized by metabolic shifts (e.g., increased oxidative phosphorylation, OXPHOS) and controlled cytokine production, enabling a balanced transition from transient pro-inflammatory responses (IL-1β, TNF-α, IL-6) toward pro-reparative phenotypes (IL-10, TGF-β, pro-angiogenic mediators). Innate immune modulation further shapes adaptive immune regulation, including Treg induction and context-dependent T helper 17 cells (Th17) modulation through dendritic cell conditioning. Structural determinants such as molecular weight, degree of sulfation, acetylation patterns, and material purity influence immune outcomes at multiple levels. The figure also highlights the critical need for endotoxin control, as contamination may result in false TLR4 activation and confound mechanistic interpretation. Downstream effects include angiogenesis, osteogenesis, collagen remodeling, and resolution of inflammation.

### 3.4. Terpenoids and Alkaloids in Immunomodulation

Beyond polyphenols, other classes of plant secondary metabolites also exhibit significant immunomodulatory properties. Terpenoids, one of the largest families of plant natural products, have been widely investigated for their anti-inflammatory and immunoregulatory activities. Several terpenoids can regulate key inflammatory signaling pathways, including NF-κB and MAPK cascades, thereby influencing macrophage activation, cytokine production, and oxidative stress responses [143,144]. For example, diterpenoids and triterpenoids derived from medicinal plants have demonstrated the capacity to attenuate excessive inflammatory signaling while promoting tissue repair processes.

Alkaloids also represent an important group of bioactive compounds with diverse immunological effects. Many plant-derived alkaloids modulate immune responses by affecting cytokine production, lymphocyte activity, and macrophage signaling pathways [145]. Certain isoquinoline and indole alkaloids have been shown to regulate inflammatory responses through modulation of NF-κB activation and oxidative stress pathways. These properties make terpenoids and alkaloids promising candidates for integration into biomaterial systems aimed at controlled immune modulation.

## 4. Macrophage Polarization and Immunomodulation: The Central Axis of Bio-Instructive Biomaterials

Across regenerative medicine and immune engineering, macrophage polarization has emerged as a unifying mechanistic axis through which biomaterials exert therapeutic effects [146,147]. Plant-based biomaterials and phytochemicals are particularly well suited to macrophage-directed immunomodulation, as their structural motifs and bioactive components directly engage immune sensing pathways and intracellular signaling networks [27,148]. Rather than acting through single-target mechanisms, these systems operate through coordinated modulation of immune recognition, metabolic reprogramming, and redox balance, collectively shaping macrophage phenotype and function [147,149].

### 4.1. M1/M2 Macrophage Polarization: A Functional Spectrum Rather than a Binary State

#### 4.1.1. Macrophage Plasticity and Phenotypic Continuum

Macrophages are among the most phenotypically plastic cells of the immune system, capable of dynamically adapting their functional state in response to microenvironmental signals [146]. This plasticity underpins their historical classification into M1 (classically activated, pro-inflammatory) and M2 (alternatively activated, anti-inflammatory and pro-reparative) phenotypes. However, contemporary immunology recognizes macrophage activation as a continuum encompassing multiple intermediate and context-dependent states rather than a rigid binary framework [150]. While the M1/M2 framework retains heuristic value, emerging single-cell transcriptomic and spatial profiling studies reveal a far more heterogeneous spectrum of macrophage activation states that remain largely unexplored in plant-based biomaterial research.

In physiological tissue repair, macrophage polarization follows a tightly regulated temporal sequence. Early after injury, macrophages adopt M1-like phenotypes that support host defense through pathogen clearance, production of pro-inflammatory cytokines, and generation of reactive oxygen species [151]. These functions are essential for eliminating microbial threats and removing damaged tissue. As repair progresses, changes in the local microenvironment, including accumulation of apoptotic cells, release of growth factors, and attenuation of inflammatory signals, drive macrophages toward M2-like phenotypes [146]. M2 macrophages promote angiogenesis, extracellular matrix remodeling, suppression of excessive inflammation, and coordination of stromal and progenitor cell activity, thereby enabling tissue regeneration [152].

Plant-based biomaterials leverage this intrinsic macrophage plasticity by providing biochemical and biophysical cues that favor timely transition from inflammatory to reparative phenotypes. Their capacity to simultaneously modulate immune signaling, oxidative stress, and metabolic state positions macrophages as primary targets of bio-instruction [147].

#### 4.1.2. Dysregulated Polarization in Pathological Contexts

In pathological conditions such as diabetic wounds, chronic inflammation, and fibrotic diseases, the physiological macrophage polarization program becomes disrupted [153,154]. The diabetic tissue microenvironment, characterized by sustained hyperglycemia, accumulation of advanced glycation end-products, excessive oxidative stress, endothelial dysfunction, and persistent inflammatory signaling, impairs macrophage phenotypic transitions [155]. As a result, macrophages become functionally “locked” in pro-inflammatory states, continuously producing cytokines (TNF-α, IL-1β, IL-6) and proteolytic enzymes that perpetuate tissue damage and inhibit regeneration [156].

This pathological macrophage persistence represents a critical therapeutic target. Biomaterial-based strategies designed to reprogram macrophages toward pro-reparative phenotypes have therefore gained substantial interest [157]. Plant-based hydrogels incorporating polysaccharides and phytochemicals are particularly effective in this context, as they provide three-dimensional matrices, sustained release of immunomodulatory agents, and mechanical cues that collectively promote M2-like polarization [158].

### 4.2. Molecular Mechanisms of Macrophage Reprogramming

#### 4.2.1. Pattern Recognition Receptor Engagement and Immune Signal Encoding

Plant-derived polysaccharides exert profound effects on macrophage behavior through engagement of pattern recognition receptors (PRRs), which function as the immune system’s primary sensors of conserved molecular motifs. Key PRRs involved include TLR2, TLR3, TLR4, and TLR9, C-type lectin receptors such as dectin-1 and dectin-2, complement receptors, and scavenger receptors including CD14 and CD36 [159,160]. Importantly, PRR activation by plant polysaccharides should not be interpreted as a simple -receptor/one-ligand interaction. Rather, immune outcomes emerge from the integration of multiple receptor-mediated signals occurring simultaneously within a defined cellular and cytokine context [161].

The specific immunological response elicited by a given polysaccharide is critically dependent on its molecular architecture. This dependence reflects a direct link between molecular structure and immune function. Rather than acting as generic ligands, plant-derived polysaccharides function as structurally programmed signals whose interpretation depends on receptor combinatorics, ligand presentation, and microenvironmental context. Parameters such as molecular weight distribution (rather than mean molecular weight alone), degree and regioselectivity of sulfation, branching frequency, and monosaccharide composition collectively determine receptor-binding preferences and downstream signaling pathways [27]. For example, β-1,3-glucans preferentially activate dectin-1–Syk signaling, whereas sulfated polysaccharides often engage multiple PRRs in parallel, including TLRs and C-type Lectin Receptors (CLRs), resulting in complex signal integration [162]. Depending on dose, exposure kinetics, and microenvironmental cues, this integration can drive either pro-inflammatory activation or immune resolution and tolerance [163].

This context dependence helps explain apparent inconsistencies in the literature, where the same named polysaccharide (e.g., fucoidan) has been reported to induce divergent immune outcomes. Such discrepancies frequently arise not from true biological contradiction, but from insufficient resolution of structural heterogeneity and experimental variables [40]. Even within a single material class, immune activity can vary substantially due to differences in molecular weight distribution, sulfation pattern, or co-extracted impurities such as proteins, polyphenols, or trace endotoxin contamination [164]. Low-level endotoxin presence can profoundly bias macrophage responses toward TLR4-mediated inflammatory activation, confounding interpretation if not rigorously controlled [165].

In addition to structural variability, dose and temporal exposure represent critical but often underappreciated determinants of immune instruction. Short, high-intensity PRR stimulation may favor inflammatory priming, whereas sustained low-level exposure can promote immune adaptation or tolerogenic responses in certain contexts [163,166]. Studies relying on single concentrations or single time-point analyses therefore provide an incomplete view of macrophage programming and limit meaningful comparison across platforms.

Collectively, these observations underscore that plant polysaccharides function as molecular “languages” rather than generic immune stimulants. Their structural features encode context-dependent immune instructions whose interpretation depends on receptor combinatorics, redox state, metabolic programming, and cytokine milieu [161,167]. Consequently, rigorous characterization of extraction methods, batch metadata, molecular weight distribution, substitution patterns, impurity profiles, and time-resolved immune readouts is not merely a technical consideration but a scientific prerequisite for reproducible structure–activity mapping and rational immune engineering [27,168].

However, an important experimental caveat must be considered when interpreting these findings. A critical methodological aspect in studies investigating the immunomodulatory effects of plant-derived polysaccharides is the potential contribution of endotoxin contamination, which may lead to artifactual activation of TLR4 signaling [169]. To address this issue, endotoxin levels should be systematically quantified using assays such as the Limulus Amebocyte Lysate (LAL) test and, when appropriate, the Monocyte Activation Test (MAT) [170]. In addition, purification and depyrogenation strategies should be implemented to minimize endotoxin content. Functional controls are also essential, including the use of polymyxin B to neutralize lipopolysaccharides and TLR4-blocking approaches to confirm pathway specificity. Comparative analyses between purified and non-purified batches, as well as validation in primary human macrophages, are recommended to ensure that the observed immunological effects are intrinsic to the biomaterial rather than due to contaminants [171].

#### 4.2.2. Redox Regulation and Metabolic Reprogramming

Reactive oxygen species represent a central convergence point linking immune signaling, metabolism, and macrophage polarization [172,173]. Excessive ROS production sustains inflammatory macrophage activation, damages mitochondrial function, and reinforces glycolytic metabolic programs characteristic of M1 phenotypes [174]. Plant-derived polysaccharides and phytochemicals counteract these processes through intrinsic antioxidant activity and activation of endogenous redox-regulatory pathways [148].

Macrophage metabolism is tightly coupled to phenotype. M1 macrophages rely predominantly on aerobic glycolysis, supporting rapid ATP generation and pro-inflammatory effector functions but producing high levels of ROS [175]. In contrast, M2 macrophages preferentially utilize oxidative phosphorylation and fatty acid oxidation, generating energy more efficiently while maintaining low oxidative stress [176]. By scavenging ROS, restoring mitochondrial integrity, and activating Nrf2-mediated antioxidant responses, plant-derived compounds facilitate the metabolic shift required for M1-to-M2 polarization [177].

This redox–metabolic coupling is particularly relevant in chronic inflammatory and diabetic settings, where oxidative stress is a dominant pathological driver [178]. While inorganic biomaterials may require doping or surface modification to achieve antioxidant effects, plant-based systems often possess intrinsic redox-regulatory capacity, conferring a distinct therapeutic advantage [179].

#### 4.2.3. Integration of PI3K/AKT, NF-κB, and MAPK Signaling Pathways

Macrophage polarization is governed by integration of multiple intracellular signaling cascades, among which PI3K/AKT, NF-κB, and MAPK pathways play central roles [180,181]. Activation of PI3K/AKT signaling promotes M2 polarization by enhancing anti-inflammatory cytokine production (IL-10, TGF-β) and suppressing NF-κB-driven inflammatory gene expression [182]. Several plant-derived polyphenols, including resveratrol, enhance PI3K/AKT signaling while simultaneously activating Nrf2-mediated antioxidant pathways [183].

NF-κB represents a key pro-inflammatory transcriptional regulator driving expression of M1-associated genes, including cytokines, chemokines, and inflammatory enzymes [184]. Plant-based compounds inhibit NF-κB activation through diverse mechanisms, including suppression of IκB kinase activity, reduction in ROS-mediated signaling, and modulation of ubiquitination-dependent pathway activation [185].

**Figure 2:**
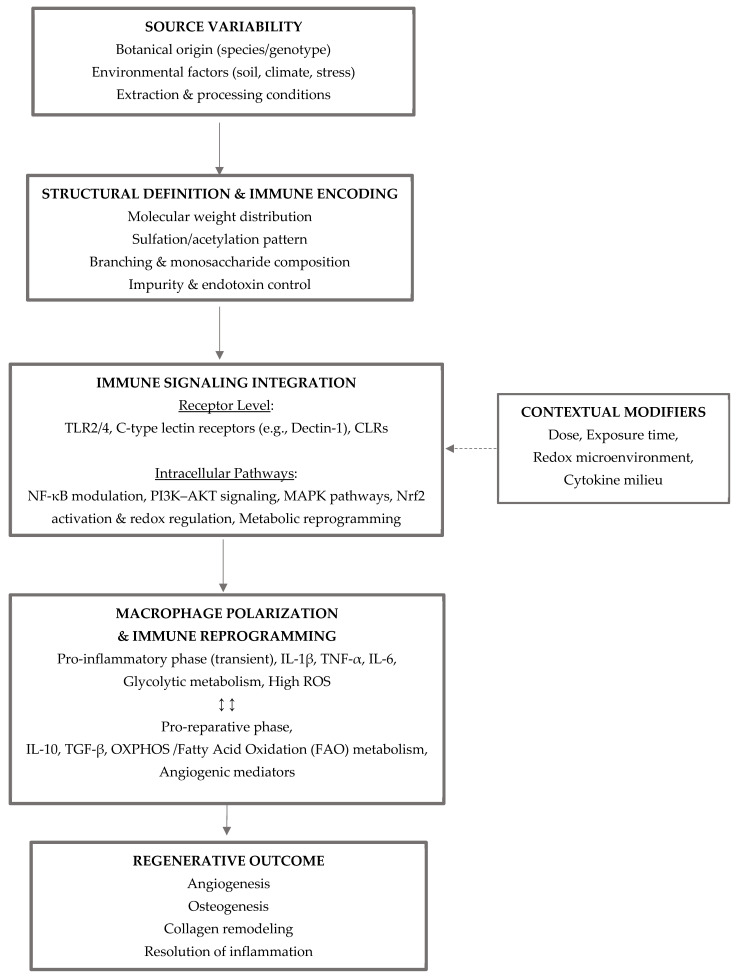
Immune-encoding framework of plant-based biomaterials.

MAPK signaling further refines macrophage phenotype. M1 macrophages exhibit dominant p38 and c-Jun N-terminal Kinase (JNK) activation, whereas M2 phenotypes are associated with Extracellular signal-Regulated Kinase (ERK)-biased signaling [186]. Plant-derived bioactive molecules selectively suppress p38 signaling while supporting ERK activation, thereby reinforcing reparative macrophage programs [187]. Such pathway-selective modulation exemplifies how plant compounds achieve immune reprogramming without global immunosuppression [188]. Figure 2 introduces a conceptual framework describing how material variability and structural features encode immune instructions that are contextually interpreted at the cellular level.

Plant-derived biomaterials translate source variability into defined structural features that encode immune-instructive signals. Molecular characteristics such as molecular weight distribution, sulfation or acetylation patterns, and purity determine engagement of pattern recognition receptors (e.g., TLR2/4, Dectin-1, CLRs) and activation of interconnected intracellular pathways, including NF-κB, PI3K–AKT, MAPK, Nrf2, and metabolic reprogramming. Signal interpretation is shaped by contextual factors (dose, exposure time, redox state, cytokine milieu), ultimately driving macrophage functional polarization along a dynamic pro-inflammatory to pro-reparative continuum. These immune responses coordinate regenerative outcomes such as angiogenesis, osteogenesis, collagen remodeling, and resolution of inflammation.

Schematic representation of how plant-derived biomaterials translate source variability into structurally encoded immune signals that are interpreted by immune cells in a context-dependent manner. Variations in botanical origin, environmental conditions, and extraction processes define key molecular features, including molecular weight distribution, substitution patterns (e.g., sulfation or acetylation), branching architecture, and impurity profiles. These structural parameters regulate the engagement of pattern recognition receptors (PRRs), such as Toll-like receptors (TLR2/4) and C-type lectin receptors, and activate interconnected intracellular signaling pathways, including NF-κB, PI3K/AKT, MAPK, and Nrf2-mediated redox regulation.

The integration of these signals, together with contextual modifiers such as dose, exposure time, and local microenvironment, drives macrophage functional polarization along a dynamic continuum from pro-inflammatory to pro-reparative states. This immune reprogramming is associated with metabolic shifts, including transitions from glycolysis to oxidative phosphorylation and fatty acid oxidation. Ultimately, these processes coordinate regenerative outcomes, including angiogenesis, osteogenesis, extracellular matrix remodeling, and resolution of inflammation. The framework highlights the importance of structural definition and experimental context in determining the immunological behavior and translational potential of plant-based biomaterials.

### 4.3. Adaptive Immune Modulation and Immune Tolerance

#### 4.3.1. Regulation of Th17/Treg Balance

Beyond innate immune regulation, plant-based biomaterials and phytochemicals exert significant influence on adaptive immunity, particularly through modulation of the Th17/Treg balance [189,190]. Th17 cells promote inflammatory responses through IL-17 production, whereas regulatory T cells suppress inflammation and maintain immune tolerance via IL-10 and Transforming Growth Factor (TGF)-β secretion [101]. Dysregulation of this balance underlies numerous chronic inflammatory and autoimmune disorders [191].

Plant-derived compounds restore immune equilibrium by promoting Treg differentiation, suppressing Th17-promoting cytokines, and attenuating inflammatory microenvironments that favor pathological immune activation [192]. When delivered via biomaterial platforms, these effects can be spatially and temporally controlled, enhancing therapeutic precision [102].

#### 4.3.2. Tolerogenic Dendritic Cells and Biomaterial-Guided Immune Reprogramming

Plant-derived bioactive molecules also influence antigen-presenting cell function, particularly by promoting differentiation of tolerogenic dendritic cells (tolDCs) [193]. These cells exhibit reduced co-stimulatory molecule expression and increased production of anti-inflammatory cytokines, driving naïve T cell differentiation toward regulatory phenotypes rather than effector responses [194].

The capacity to engineer immune tolerance through biomaterial-guided dendritic cell programming represents a critical frontier in regenerative medicine and immunotherapy [194]. Plant-based biomaterials, by integrating innate and adaptive immune modulation within a single platform, offer a powerful and biologically congruent approach to immune reprogramming [195].

Overall, these findings indicate that plant-derived biomaterials should not be regarded as uniform bioactive systems, but rather as structurally encoded immunomodulators whose biological effects are critically determined by fine molecular features. Establishing reproducible structure–activity relationships therefore represents a central requirement for their rational design and clinical translation.

## 5. Plant-Based Biomaterials in Tissue Engineering: Tissue-Specific Applications and Mechanistic Considerations

The regenerative outcome of biomaterial-based therapies is strongly influenced by tissue-specific immune requirements and microenvironmental constraints [19]. Although core immunomodulatory mechanisms, including macrophage polarization, redox regulation, and cytokine signaling, are conserved across tissues, their relative contribution and temporal dynamics vary substantially depending on the local biological context [194]. Within this framework, plant-derived biomaterials offer a unique advantage, as their structural features—including molecular weight distribution, degree of substitution, and chemical composition—enable context-dependent modulation of immune responses. These materials should therefore be considered not as passive scaffolds, but as structurally encoded immunomodulatory systems capable of directing tissue-specific repair processes. In addition to marine and medicinal plant polysaccharides, structurally oriented plant polymers such as cellulose, starch, and pectin (Section 2.4) are increasingly explored for regenerative applications, where their biocompatibility is complemented by emerging roles in immune signaling modulation [196].

This section examines the application of plant-derived biomaterials and phytochemicals in wound healing, bone regeneration, and cartilage and neural tissue engineering, with emphasis on the interplay between material structure, immune regulation, and tissue-specific design strategies. As summarized in Table 1, these biomaterials have been investigated across multiple regenerative contexts, including skin, bone, cartilage, and neural tissues, where they integrate structural support with immunomodulatory signaling to coordinate inflammatory resolution and tissue regeneration in a context-dependent manner.

### 5.1. Wound Healing: From Acute Repair to Chronic Wound Management

#### 5.1.1. Immune Orchestration During Physiological Wound Healing

Cutaneous wound healing is a highly coordinated, multistep biological process classically divided into three overlapping phases: inflammation (approximately days 0–7), proliferation (days 7–21), and remodeling (weeks to months) [197]. Across all phases, immune regulation serves as the central organizing principle, integrating inflammatory signaling, cellular recruitment, tissue repair, and resolution processes [103].

During the inflammatory phase, rapid hemostasis is followed by infiltration of neutrophils and macrophages that eliminate pathogens, clear necrotic debris, and initiate immune signaling through cytokine and chemokine release [198]. Reactive oxygen species play a dual role at this stage, contributing to antimicrobial defense while also functioning as secondary messengers in immune signaling [199]. Excessive suppression of inflammation at this stage compromises healing, underscoring the need for balanced immune modulation rather than blanket anti-inflammatory strategies [198].

The proliferative phase is characterized by a shift toward reparative immune activity. M2-like macrophages become predominant, promoting angiogenesis, fibroblast proliferation, granulation tissue formation, and extracellular matrix deposition [152]. Growth factors such as vascular endothelial growth factor (VEGF), basic fibroblast growth factor (bFGF), and transforming growth factor-β (TGF-β) orchestrate vascularization and matrix synthesis [21]. The remodeling phase involves collagen realignment and crosslinking, maturation of the vascular and neural networks, and restoration of functional barrier properties.

Plant-based biomaterials are particularly effective in supporting this dynamic immune choreography, as they can provide phase-appropriate immunomodulatory cues while simultaneously offering structural support.

#### 5.1.2. Advanced Immunomodulatory Hydrogel Systems for Wound Healing

Among wound healing biomaterials, hydrogels have emerged as particularly powerful platforms for immune regulation due to their high water content, tunable mechanical properties, and capacity for controlled release of bioactive agents [200,201]. Plant-derived polysaccharide hydrogels, used alone or in combination with phytochemicals, enable precise modulation of inflammatory, oxidative, and reparative pathways while simultaneously providing a permissive structural matrix for tissue regeneration [104].

Modern wound dressings now encompass a broad spectrum of formats, including hydrogels, aerogels, electrospun nanofibrous membranes, sponges, microneedle arrays, and 3D-printed constructs [202]. Increasingly, these systems are designed not as passive barriers but as active immunoregulatory devices capable of targeting mitochondrial function, redox homeostasis, autophagy, ferroptosis, and macrophage phenotype [198].

Particularly advanced hydrogel designs incorporate spatiotemporally controlled release profiles aligned with the physiological phases of wound healing [203]. Antimicrobial agents are preferentially delivered during the early inflammatory phase, antioxidant compounds are released throughout healing to regulate reactive oxygen species levels, and pro-reparative factors, such as growth factors or M2-polarizing phytochemicals, are deployed during the proliferative phase [138]. This phase-adaptive strategy more closely mimics endogenous healing processes and has demonstrated superior outcomes compared to conventional passive dressings [13].

Stimuli-responsive hydrogels further enhance therapeutic precision. For example, matrix metalloproteinase-responsive systems exploit elevated Matrix Metalloproteinase (MMP)-9 activity characteristic of chronic and diabetic wounds to trigger on-demand release of immunomodulatory cargo, including M2 macrophage-derived extracellular vesicles (EVs) [204]. Such conditional delivery limits off-target exposure and ensures that immune-modulating signals are deployed selectively under pathological inflammatory conditions [21].

#### 5.1.3. Plant-Derived Extracellular Vesicles in Wound Repair: Isolation, Characterization, and Translational Standardization Challenges

A rapidly emerging area of interest is the use of plant-derived extracellular vesicle–like nanoparticles (EV-like nanoparticles) as immunomodulatory agents in wound healing [105]. EV-like nanoparticles isolated from fruits and medicinal plants, such as grapes, lemons, and various herbs, contain complex cargos of bioactive lipids, proteins, metabolites, and nucleic acids reflective of their plant origin and physiological function [205]. Preclinical studies indicate that plant-derived EV-like nanoparticles can modulate macrophage polarization and inflammatory signaling in experimental models, promoting the transition from pro-inflammatory to pro-reparative phenotypes while also supporting endothelial migration and angiogenic responses in wound healing contexts [206,207,208]. These effects are particularly pronounced in diabetic wound models, where chronic inflammation, oxidative stress, and impaired immune resolution severely compromise physiological healing trajectories [209].

When incorporated into polysaccharide-modified Gelatin Methacryloyl (GelMA) or other plant-based hydrogel systems, plant-derived EV-like nanoparticles integrate biological signaling capacity with mechanical and structural support, yielding synergistic improvements in wound closure, granulation tissue formation, and neovascularization. Conceptually, these platforms bridge traditional plant-based therapeutics with contemporary nanomedicine and biomaterials engineering, offering a scalable, biologically congruent, and immunologically active strategy for wound repair [210].

Here, we use the term EVs as an umbrella definition, in line with current International Society for Extracellular Vesicles (ISEV) recommendations, to reflect the heterogeneous nature of vesicular populations isolated from plant tissues. While several studies refer to these preparations as “exosomes”, rigorous demonstration of an endosomal biogenesis pathway remains limited in plant systems. We therefore adopt EVs as the most accurate and conservative terminology, retaining the term “exosomes” only when explicitly supported by the original characterization [211]. Despite these promising biological effects, several methodological and translational aspects require careful consideration. Isolation approaches typically include differential ultracentrifugation, density gradient separation, size-exclusion chromatography (SEC), and polymer-based precipitation methods, each differing in yield, purity, and scalability [212,213]. Comprehensive characterization is essential and generally involves particle size and concentration analysis by nanoparticle tracking analysis (NTA), morphological assessment by transmission electron microscopy (TEM) or cryo-TEM, and molecular profiling of cargo components through proteomics, small RNA sequencing, and lipidomics [214,215].

Compared to mammalian exosomes, plant-derived EVs present distinct features, including their origin from cell wall-containing systems, differences in cargo composition, and the lack of universally accepted molecular markers, which complicates their classification and comparison across studies. Moreover, the mechanisms of biogenesis and subtype definition remain less clearly established [216,217].

From a translational perspective, several challenges still hinder their clinical development, including limited comparability between studies, co-isolation of non-vesicular components, low scalability of current isolation protocols, and the absence of standardized, Good Manufacturing Practice (GMP)-compliant production workflows and quality control criteria. Variability in isolation techniques further contributes to variability in EV preparations and represents a major barrier to clinical translation. These limitations highlight the need for harmonized methodologies and robust characterization pipelines to ensure reproducibility and facilitate clinical implementation [218].

### 5.2. Bone Tissue Engineering and Osteoimmunology

Bone regeneration is increasingly understood through the lens of osteoimmunology, which recognizes immune cells, particularly macrophages, as central regulators of osteogenesis and bone remodeling [102,219]. Rather than suppressing inflammation indiscriminately, successful bone regeneration requires finely tuned immune activation that supports osteoblast differentiation, angiogenesis, and matrix mineralization while avoiding chronic inflammatory signaling [220].

Plant-based polysaccharide biomaterials are well suited for bone tissue engineering due to their structural resemblance to native extracellular matrix components and their intrinsic immunomodulatory capacity [221]. As summarized in Table 1, several plant-derived biomaterials, including fucoidan-based systems, ulvan-derived scaffolds, and polyphenol-containing biomaterials, have been investigated in the context of bone regeneration, where their immunomodulatory activity can promote osteogenic signaling and vascularization. By promoting pro-reparative macrophage phenotypes, often described as M2-like states, these materials enhance the secretion of osteogenic and angiogenic mediators [147]. Similar osteo-immunomodulatory effects of marine and plant-derived polysaccharides have also been described in recent biomaterials studies [82,222].

Composite systems, such as chitosan-based nanocomposite hydrogels, exemplify the potential of integrating natural polymers with inorganic nanomaterials to achieve mechanical robustness without sacrificing biological activity [223]. These scaffolds provide both physical cues and immune-instructive signals, guiding macrophages toward phenotypes that actively support osteogenesis rather than fibrotic encapsulation [224]. Comparable strategies using polysaccharide-based nanocomposites and bioactive nanoparticles have recently been explored to further enhance osteogenic differentiation and bone repair [225].

Phyto-nanoparticles, nanoparticles synthesized using plant extracts as reducing and stabilizing agents, represent an additional innovative strategy in this context [226]. These systems inherit antioxidant and immunomodulatory properties from their plant precursors while enhancing osteogenic signaling pathways, including Runt-related transcription factor 2 (RUNX2) and Osterix activation [227]. Recent studies have shown that plant-mediated nanoparticle synthesis may improve osteogenic differentiation and reduce oxidative stress in bone regeneration models [228]. Despite promising preclinical outcomes, challenges related to batch reproducibility, scale-up, and regulatory classification remain substantial and must be addressed before clinical translation can be realistically pursued [168].

### 5.3. Cartilage and Neural Tissue Engineering

Cartilage regeneration presents unique challenges due to limited intrinsic healing capacity and sensitivity to inflammatory damage [229]. Glycopeptide hydrogels constructed through dynamic covalent bonding between polysaccharides and peptides closely mimic the viscoelastic and biochemical properties of native cartilage extracellular matrix [230]. Their self-healing and stimuli-responsive characteristics allow them to withstand mechanical loading while providing sustained immunomodulatory signaling necessary to suppress inflammation and support chondrogenesis [231].

Plant-derived polysaccharides and related biomaterials have also been widely explored in cartilage tissue engineering because of their biocompatibility and capacity to regulate inflammatory responses. As indicated in Table 1, materials such as alginate-based hydrogels and other polysaccharide scaffolds have been investigated in cartilage regeneration, where their viscoelastic properties and ability to encapsulate cells or growth factors make them particularly suitable for cartilage repair applications [25].

Neural tissue engineering imposes even stricter immunological constraints, as excessive inflammation and microglial activation can severely impair neural regeneration [231]. Seaweed-derived polysaccharides offer particular advantages in this context, as their structural and biochemical properties resemble neural extracellular matrix components while delivering inherent anti-inflammatory and antioxidant signals [232]. Marine polysaccharides such as alginate and other seaweed-derived polymers have therefore been explored as biomaterials capable of modulating neuroinflammation and supporting neural repair processes [17,18]. By attenuating neurotoxic microglial activation and oxidative stress, plant-based materials create permissive environments for neuronal survival and axonal regrowth [233].

Taken together, these studies highlight the broad range of tissue engineering applications of plant-derived biomaterials, including skin, bone, cartilage, and neural regeneration, as summarized in Table 1.

## 6. Translational Challenges and Pathways Toward Clinical Application

The scientific rationale supporting plant-based immunomodulatory biomaterials is increasingly robust, grounded in advances in immune signaling, macrophage plasticity, and regenerative immunology [154,168,233]. However, translation into clinical practice has progressed more slowly than mechanistic advances would predict. This discrepancy does not reflect a lack of therapeutic promise. Rather, it arises from a convergence of unresolved challenges such as spanning material standardization, immune validation, manufacturing scalability, and regulatory positioning [103,113,114]. The most significant regulatory barriers include intrinsic material variability, lack of standardized structure–function definitions, and ambiguity in regulatory classification, particularly for systems that combine structural and immunomodulatory functions.

Plant-derived biomaterials are intrinsically heterogeneous, multicomponent systems in which biological activity often emerges from synergistic and network-level interactions rather than single defined molecules [10,11,12,113]. Such compositional complexity complicates conventional development paradigms built upon chemical uniformity and clearly defined active ingredients [113,114]. Moreover, the dual identity of these platforms, as both structural scaffolds and active immunomodulators, places them at the interface between structural biomaterials and immunologically active agents, increasing developmental complexity [1,3,103].

Beyond regulatory positioning, translational barriers include insufficient structure–function mapping in natural product research [113,114], inadequate control of endotoxin contamination, an established confounder in biomaterials immunology [104,174], overreliance on simplified in vitro macrophage polarization models [155,191], and limited use of clinically relevant immune validation systems, given the well-documented discrepancies between preclinical models and human inflammatory responses [177]. Without systematic management of biological variability and explicit linkage between defined chemical parameters and validated bioactivity endpoints, reproducibility and translational confidence remain compromised [103,113].

Overcoming these barriers demands a paradigm shift from exploratory bioactivity reporting toward reproducible immune-engineering strategies. Standardized sourcing and rigorous phytochemical characterization [14,15,16], comprehensive physicochemical profiling [87,232], validated multiparametric immune assays that extend beyond static polarization markers [155,187], and integration of tissue-relevant experimental models [200,230] must be embedded early in development. The following sections critically analyze these translational constraints and delineate strategic pathways capable of converting mechanistic promise into clinically reliable, scalable, and biologically coherent immunomodulatory therapies. Key translational stages, critical quality attributes, and regulatory considerations are summarized in Table 2.

Summary of critical development stages from raw material sourcing to regulatory classification, outlining required actions, critical quality attributes (CQAs), and representative literature references. The framework integrates standardization strategies, endotoxin control, GMP considerations, and regulatory pathway alignment necessary to support clinical translation.

### 6.1. Current Level of Clinical Evidence

Among the plant-derived biomaterials discussed, the strongest clinical evidence currently supports acemannan-containing topical formulations and Aloe-derived products for wound healing and skin repair, although variability in study design and product standardization remains a limitation [79,80]. Fucoidan has shown emerging translational potential, primarily in nutraceutical and adjunct biomedical applications, but clinical evidence is still limited [40]. Alginate is widely used in clinical settings, particularly in wound dressings and encapsulation systems, although its immunomodulatory activity is less directly established compared to its structural and functional roles [63]. In contrast, ulvan, LBP, and plant-derived extracellular vesicles remain largely at the preclinical stage, with limited or no robust clinical validation [61,71,218]. Despite these advances, the translation of plant-derived biomaterials into clinically standardized products remains limited and faces several critical challenges, including regulatory classification, reproducibility, and manufacturing variability, which are discussed below.

### 6.2. Advanced Delivery Systems and Bioavailability Enhancement

A central limitation of many plant-derived bioactive compounds is their unfavorable pharmacokinetic profile, characterized by poor aqueous solubility, limited membrane permeability, rapid metabolic degradation, and low systemic or local bioavailability [94,105,118,205,206]. Advanced delivery systems are therefore essential to unlock the full therapeutic potential of plant-based immunomodulatory biomaterials [239,240].

Among these strategies, phytosome technology, based on the complexation of phytochemicals with phospholipids, has demonstrated particular promise in improving stability, membrane interaction, and tissue uptake of polyphenols and other bioactive small molecules [241,242]. Compared to conventional encapsulation approaches, phytosomes promote closer molecular association between the active compound and lipid carrier, enhancing both bioavailability and sustained release [243].

Polysaccharide-based nanoparticles, injectable hydrogels, and nanocomposite scaffolds further enable localized and controlled delivery, minimizing systemic exposure while maintaining therapeutically relevant concentrations within target tissues [239]. Importantly, delivery platforms also modulate immune outcomes by shaping release kinetics, spatial gradients, and co-presentation of multiple bioactive cues [102,157].

Despite these advances, cross-study comparability remains limited. Differences in extract composition, phospholipid ratios, formulation protocols, and pharmacokinetic endpoints complicate interpretation and translation [234,244]. Harmonized characterization strategies and clinically meaningful dosing frameworks are therefore essential to ensure that delivery innovations translate into reproducible therapeutic benefit [235,236].

Control of biomass variability at scale requires integrated management of upstream and downstream variables, including authenticated source material, controlled cultivation/harvest conditions, predefined chemotype specifications, standardized extraction Standard Operating Procedures (SOPs), orthogonal analytical fingerprinting, and mechanism-linked potency assays as release criteria.

### 6.3. Ethical and Regulatory Considerations in Plant-Based Biomaterial Development

#### 6.3.1. Ethnopharmacology, Indigenous Knowledge, and Ethical Translation

Ethnopharmacology occupies a critical position at the interface between traditional medical knowledge and contemporary biomedical research, offering a historically informed framework for identifying biologically active plant-derived compounds [14]. For plant-based biomaterials, ethnopharmacological knowledge provides valuable insights into bioactivity, safety, and modes of administration that often predate modern experimental system [15]. However, meaningful integration of this knowledge into biomaterials research remains limited and uneven [237,238].

A persistent challenge lies in the asymmetry between scientific extraction of traditional knowledge and equitable recognition or compensation of indigenous communities. While international frameworks such as the Nagoya Protocol and World Health Organization (WHO) guidelines formally recognize the rights of indigenous peoples over genetic resources and associated traditional knowledge, their implementation remains inconsistent across jurisdictions [245,246]. In practice, benefit-sharing agreements are frequently complex, under-enforced, or absent, increasing the risk of biopiracy and ethical misconduct [247,248].

From a translational perspective, ethical engagement with ethnopharmacology must extend beyond formal legal compliance. Responsible development of plant-based biomaterials requires transparent documentation of knowledge provenance, culturally informed consent processes, and long-term benefit-sharing mechanisms that include not only financial returns but also capacity building, technology transfer, and access to resulting therapies [245,248]. Case studies such as Euphorbia peplus-derived ingenol mebutate and Cordyceps-based therapeutics illustrate that ethically responsible pathways from traditional use to clinical validation are achievable when scientific rigor is coupled with social accountability [249,250].

Importantly, ethical considerations also intersect with scientific robustness. Traditional preparation methods often differ substantially from laboratory extraction protocols, potentially altering bioactive profiles and immunological outcomes [238,251]. Failure to acknowledge and systematically investigate these differences can lead to misinterpretation of efficacy, safety, and mechanism of action [238,244].

#### 6.3.2. Standardization, GMP Compliance, and Regulatory Pathways

Regulatory approval remains one of the most significant bottlenecks in the clinical translation of plant-based biomaterials. Unlike synthetic polymers or single-molecule drugs, plant-derived materials are intrinsically variable systems. Phytochemical composition is influenced by botanical species, genotype, geographic origin, soil composition, climate, harvest timing, post-harvest processing, and storage conditions. This biological variability presents a fundamental challenge to regulatory frameworks that are predicated on chemical uniformity and batch-to-batch reproducibility [235,246].

Standardization therefore represents not merely a regulatory requirement, but a central scientific challenge. Advanced analytical tools, such as high-performance liquid chromatography, mass spectrometry, and nuclear magnetic resonance, enable increasingly detailed chemical characterization of plant extracts and polysaccharides [251,252]. However, chemical fingerprinting alone is insufficient. Regulatory acceptance requires demonstration that defined chemical parameters correlate reliably with biological function, necessitating rigorous structure–activity relationship studies and validated bioassays [64,81,236].

GMP compliance further complicates translation. Many traditional extraction and processing methods lack the traceability, documentation, and quality control demanded for clinical-grade manufacturing. Scaling up production while maintaining biological activity and reproducibility often requires substantial modification of extraction protocols, which can in turn alter immunomodulatory properties. This creates tension between preserving bioactivity and achieving regulatory compliance [235,253].

Another unresolved challenge concerns regulatory classification. Plant-based biomaterials often occupy ambiguous positions between medical devices, biologics, and combination products. This regulatory ambiguity can slow approval processes and increase development costs, particularly when immunomodulatory activity blurs the distinction between structural support and pharmacological function. Clearer regulatory pathways and harmonized international guidelines will be essential to accelerate clinical translation without compromising safety [253,254,255]. Early dialogue with regulatory agencies to define product classification and evidentiary requirements may significantly reduce downstream development risk, particularly for platforms positioned at the interface between medical devices and biologically active products.

### 6.4. Evidence Quality, Reproducibility, and “Regulatory Readiness”: A Critical Appraisal

A major limitation across the plant-based biomaterials literature is the pronounced heterogeneity of experimental models, material definitions, and biological endpoints, which substantially complicates both mechanistic interpretation and translational decision-making [234,244]. A large proportion of studies rely on in vitro macrophage assays performed in immortalized cell lines, often using supraphysiological concentrations and short exposure times, with limited validation in human primary cells, disease-relevant models, or clinically realistic dosing scenarios [168,256]. Therefore, reported immunomodulatory effects frequently lack a clear path toward clinical extrapolation. Future studies should prioritize integration of primary human immune cells, organoid-based inflammatory models, and humanized systems to improve predictive validity and reduce translational attrition.

An additional and pervasive challenge concerns insufficient material definition. Plant-derived extracts and polysaccharides are often described using generic labels without adequate batch metadata, detailed chemical fingerprinting, or rigorous impurity and endotoxin control. Given the intrinsic biological variability of plant sources, this lack of definition makes it difficult to attribute observed immune effects to specific molecular features and represents a major barrier to reproducibility [236,246]. In many cases, apparent discrepancies between studies can be traced to differences in botanical origin, harvest conditions, extraction protocols, or post-processing steps rather than to true biological inconsistency [238,251].

Improving translational relevance therefore requires a shift toward systematic standardization across the entire development pipeline. At the source level, botanical authentication, cultivar or genotype documentation, geographic origin, seasonality, and harvesting and storage conditions should be explicitly defined, as these parameters directly influence chemical composition and bioactivity [246]. Extraction and purification processes must similarly be fixed and documented, including solvent systems, temperature and time parameters, and purification strategies, with deliberate justification provided when co-extracted components such as proteins or polyphenols are retained as part of the functional material [251].

From a materials science perspective, regulatory readiness depends on identification and control of critical quality attributes. These include molecular weight distribution rather than average values alone, substitution patterns such as sulfation or acetylation degree and regioselectivity, monosaccharide composition and branching indices, impurity and endotoxin thresholds, and stability metrics under relevant storage and physiological conditions [257,258]. Chemical characterization, however, is not sufficient in isolation. Defined chemical parameters must be explicitly linked to biological potency through validated bioactivity assays [236].

Accordingly, bioactivity assessment should move beyond single cytokine measurements toward multiparametric and time-resolved immune profiling. Functional macrophage assays, such as efferocytosis capacity, oxidative burst regulation, and secretion of pro-angiogenic mediators, provide more meaningful insight into regenerative potential than static polarization markers alone [259]. These assays should be complemented by polarization signatures assessed over time, pattern recognition receptor engagement proxies with appropriate impurity controls, and tissue-relevant co-culture systems that better reflect in vivo immune–tissue crosstalk [260,261].

Manufacturing considerations further constrain clinical translation. Scaling plant-based biomaterials to clinical-grade production requires development of robust, documented, and reproducible standard operating procedures compatible with good manufacturing practice. Cleaning and sterilization methods must be validated to preserve polysaccharide integrity or extracellular vesicle functionality, while release criteria should be directly tied to both critical quality attributes and validated potency assays. Stability-indicating methods are also essential to establish shelf-life and ensure consistent performance over time [253].

Finally, early and explicit regulatory classification is crucial. Plant-based immunomodulatory biomaterials often occupy ambiguous positions at the interface of medical devices, drugs or biologics, and combination products. Delayed or unclear classification increases development costs and regulatory risk, as evidence requirements, clinical endpoints, and post-market obligations differ substantially across regulatory pathways. Proactive alignment with regulatory agencies to define classification and evidentiary expectations can therefore significantly accelerate translation [255].

Taken together, these considerations underscore that successful clinical translation of plant-based biomaterials does not require elimination of biological variability, but rather its systematic management. Variability must be engineered into control through defined quality attributes, validated bioactivity assays, and early regulatory alignment [255]. Only through such integrated approaches can the field move from promising preclinical observations toward reproducible, clinically reliable, and regulatory-compliant immunomodulatory therapies [234].

In Table 3 are showed minimum requirements for chemical characterization, endotoxin testing, immune profiling, mechanistic validation, human-relevant modeling, extracellular vesicle characterization (when applicable), and batch comparability. The table outlines key methodological benchmarks intended to improve reproducibility, mechanistic clarity, and alignment with regulatory expectations in the development of plant-based immunomodulatory biomaterials.

### 6.5. Immunotoxicity and Risks of Immune Overactivation

An additional and often underexplored aspect in the translational development of plant-based immunomodulatory biomaterials concerns their potential immunotoxicity and the risk of unintended immune overactivation. While these systems are designed to actively engage immune pathways, this same bioactivity may lead to excessive or dysregulated responses if not properly controlled.

In particular, overstimulation of pattern recognition receptors, especially TLR2- and TLR4-mediated pathways, may result in sustained pro-inflammatory signaling, elevated cytokine production, and impaired resolution of inflammation. Such effects are highly context-dependent and may be exacerbated by high local concentrations, prolonged exposure, or structurally heterogeneous material preparations. In pathological settings, uncontrolled immune activation may contribute to chronic inflammation, fibrotic responses, or delayed tissue regeneration [155,167].

These risks are closely linked to material-intrinsic properties, including molecular weight distribution, degree and pattern of chemical substitution, and the presence of co-extracted impurities. As discussed above, endotoxin contamination represents a particularly critical confounding factor, as even trace levels may induce strong TLR4-mediated responses and obscure the true immunological activity of the biomaterial [164,169].

In addition, dose and exposure kinetics play a central role in determining whether immune activation remains beneficial or becomes detrimental. While transient and controlled stimulation may promote macrophage reprogramming toward pro-reparative phenotypes, sustained or excessive stimulation may bias immune cells toward persistent pro-inflammatory states, ultimately impairing tissue regeneration [163,166].

For these reasons, careful evaluation of immunotoxicity and immune safety profiles should be integrated early in biomaterial development. This includes standardized endotoxin testing, dose–response studies, and multiparametric immune profiling using primary human immune cells and tissue-relevant models [167,229]. Such approaches are essential to ensure that plant-based biomaterials promote controlled immune regulation rather than uncontrolled immune activation, thereby supporting both safety and translational reliability.

## 7. Conclusions and Future Perspectives

Plant-based biomaterials represent a rapidly evolving class of bio-instructive platforms that blur traditional boundaries between structural materials and therapeutics. By actively engaging immune systems, particularly via macrophage reprogramming, redox regulation, and coordinated innate–adaptive crosstalk, these materials can create pro-reparative microenvironments that support tissue regeneration across multiple biomedical contexts [102,146,157,173,223].

Nevertheless, clinical translation remains limited not primarily due to lack of promising mechanisms, but due to gaps in reproducibility, standardization, and regulatory readiness. Intrinsic source variability, incomplete structure–activity relationships, inconsistent potency assays, and ambiguous regulatory classification frequently prevent otherwise compelling platforms from advancing beyond preclinical stages [234,236,255].

Future progress will require (i) mechanistic clarity linking defined structural features to immunological outcomes [64,81,157]; (ii) design principles that treat immune modulation as context-dependent orchestration rather than generic suppression [150,223]; (iii) delivery systems enabling phase-appropriate, tissue-specific spatiotemporal control [104,201,202]; (iv) rigorous, clinically meaningful validation strategies and endpoints [234,244,256]; (v) ethically grounded development pipelines that ensure sustainable sourcing and equitable benefit-sharing [246,247,248]. The future of plant-based immunomodulatory biomaterials will depend less on the discovery of new bioactive compounds and more on the ability to engineer reproducibility, regulatory clarity, and context-specific immune control into clinically reliable platforms [235,236].

## Figures and Tables

**Table 1:** Structural and immunological characteristics of major plant-based biomaterials.

Material	Key Structural Determinants	Main PRRs/ Targets	Dominant Immune Effects	Tissue Engineering Applications	Evidence Level	Key References
Fucoidan	Sulfated fucose-rich polysaccharide; molecular weight distribution; degree and regioselective sulfation	TLR2, TLR4, scavenger receptors, selectins	Anti-inflammatory; macrophage polarization (M2-like); angiogenesis support	Wound healing, bone regeneration, hydrogels	In vitro, in vivo, limited clinical evidence	[31,33,38,40,43,82]
Alginate	Mannuronic/guluronic acid (M/G ratio); molecular weight; purity (endotoxin content)	TLR2, TLR4 (purity-dependent)	Immune shielding; modulation of foreign body response; fibrosis control (when purified)	Injectable hydrogels, cell encapsulation, cartilage and wound repair	Extensive in vivo	[20,51,54,55]
Carrageenan	Sulfated galactans (κ, ι, λ isoforms); sulfation pattern and density	TLR4 (context-dependent)	Context-dependent pro- or anti-inflammatory effects; immune cell recruitment	Inflammatory models, antiviral and wound systems	In vitro, in vivo	[56,57,58,59]
Ulvan	Sulfated heteropolysaccharide (rhamnose, xylose, uronic acids); branching and charge density	TLR-mediated pathways (proposed), adhesion molecules	Antioxidant; immunomodulatory; macrophage activation	Tissue engineering scaffolds, wound healing	Preclinical	[60,61,62,63]
LBP	Heterogeneous polysaccharide–protein complexes; branching and monosaccharide composition	TLR2, TLR4	Macrophage activation; cytokine modulation; microbiota-mediated immune effects	Oral immunomodulation, systemic immune regulation	Preclinical	[66,68,70,71,72]
Acemannan	Acetylated β-(1→4)-mannan; degree of acetylation	TLR4	Macrophage activation; wound healing; fibroblast stimulation	Wound healing, regenerative dressings	Preclinical + topical clinical	[74,76,78,79,80]
Curcumin	Hydrophobic polyphenol; pleiotropic molecular interactions; low bioavailability	PRR-independent; NF-κB, MAPK, PI3K/AKT (indirect modulation)	Anti-inflammatory; antioxidant; immunometabolic regulation	Bone regeneration, wound healing, nanocarriers	Extensive preclinical	[96,97,98,99,100]
Plant-derived EV-like nanoparticles	Lipid bilayer vesicles; mRNA, protein, and metabolite cargo	Endocytosis; PRR-independent and indirect signaling pathways	Modulation of inflammation; macrophage reprogramming; tissue repair	Wound healing, regenerative nanomedicine	Early-stage preclinical	[101,102,103,104,105]

**Table 2:** Translational and regulatory framework for plant-based immunomodulatory biomaterials.

Development Stage	Key Actions	Critical Quality Attributes (CQAs)	Key References
Raw Material Sourcing	Botanical authentication; GACP compliance; traceability of origin	Species identity; genotype; geographic origin; harvest timing; environmental conditions	[14,15,16]
Extraction & Processing	Standardized extraction protocols; solvent control; temperature/time optimization	Molecular weight distribution; sulfation/acetylation degree; compositional consistency	[86]
Purification	Endotoxin removal; impurity reduction; removal of co-extracted components (if required)	Endotoxin thresholds (LAL/MAT); residual solvents; protein/polyphenol contamination	[155,164,232]
Chemical Characterization	Advanced analytical profiling (HPLC, MS, NMR); batch comparison	Structural fingerprint; substitution pattern; monosaccharide composition; branching	[86,234,235]
Biological Testing	Multiparametric immune assays; dose–response and time-course studies	Mechanism-linked potency assays; cytokine panels; macrophage functional assays	[139,167,232]
Preclinical Validation	Use of primary human cells; tissue-relevant models; in vivo validation	Translational relevance; reproducibility across models; immune safety profile	[167,229]
GMP Manufacturing	SOP development; scale-up validation; batch reproducibility	Stability; batch-to-batch consistency; validated release criteria	[231,236]
Regulatory Classification	Early regulatory engagement; classification strategy (device/biologic/combination)	Defined mechanism of action; risk assessment; regulatory pathway alignment	[236,237,238]

**Table 3:** Recommended preclinical and analytical standards for regulatory readiness.

Category	Minimum Requirements	Scientific Rationale	Key References
**Chemical** **Characterization**	Full compositional profiling (HPLC, MS, NMR); MW distribution; substitution degree and pattern	Establish structure–activity relationships and ensure reproducibility	[86,234,235]
**Structural** **Definition**	Sulfation/acetylation mapping; monosaccharide composition; branching analysis	Link molecular architecture to immune signaling pathways	[81,82,84]
**Endotoxin Control**	LAL assay or monocyte activation test (MAT); defined thresholds	Prevent false TLR4-mediated immune activation	[155,164,169]
**Immune Profiling**	Multiplex cytokine analysis; time-resolved studies; macrophage functional assays	Capture dynamic immune responses beyond static M1/M2 markers	[139,167,232]
**Mechanistic** **Validation**	PRR blocking experiments; receptor-binding assays; signaling pathway analysis	Demonstrate causality between structure and immune response	[27,148,159,160]
**Human-Relevant Models**	Primary human macrophages; co-culture systems; organoids	Improve predictive validity and translational relevance	[167,229]
**Batch Consistency**	Cross-batch chemical and biological comparison	Ensure reproducibility and regulatory compliance	[231,236]
**Stability Testing**	Shelf-life studies; degradation profiling under physiological conditions	Guarantee consistent performance over time	[236]
**EV Characterization** **(if applicable)**	Size distribution; marker profiling; purity assessment (ISEV guidelines)	Standardization of extracellular vesicle research	[211,214,215]

## Data Availability

No new data were created.
